# International Perspective on Health Literacy and Health Equity: Factors That Influence the Former Soviet Union Immigrants

**DOI:** 10.3390/ijerph17062155

**Published:** 2020-03-24

**Authors:** Uliana Kostareva, Cheryl L. Albright, Eva-Maria Berens, Diane Levin-Zamir, Altyn Aringazina, Maria Lopatina, Luba L. Ivanov, Tetine L. Sentell

**Affiliations:** 1 School of Nursing and Dental Hygiene, University of Hawai’i at Mānoa, Honolulu, HI 96822, USA; cherylal@hawaii.edu; 2 Interdisciplinary Center for Health Literacy Research, Bielefeld University, 33699 Bielefeld, Germany; eva-maria.berens@uni-bielefeld.de; 3 Department of Health Education and Promotion, Clalit Health Services, School of Public Health, University of Haifa, Haifa 31000, Israel; diamos@zahav.net.il; 4 Kazakhstan School of Public Health, Medical University, Almaty 050000, Kazakhstan; altyn.aringazina@gmail.com; 5 National Medical Research Center for Therapy and Preventive Medicine of the Ministry of Health of the Russian Federation, 101000 Moscow, Russia; MLopatina@gnicpm.ru; 6 Chamberlain College of Nursing, Chamberlain University, Downers Grove, 60515 IL, USA; LIvanov@chamberlain.edu; 7 Office of Public Health Studies, University of Hawai’i at Mānoa, Honolulu, HI 96822, USA; tsentell@hawaii.edu

**Keywords:** immigrant, migrant, refugee, Soviet, Russian-speaking, access to care, healthcare system

## Abstract

Among the world’s 272 million international migrants, more than 25 million are from the former Soviet Union (FSU), yet there is a paucity of literature available about FSU immigrants’ health literacy. Besides linguistic and cultural differences, FSU immigrants often come from a distinct healthcare system affecting their ability to find, evaluate, process, and use health information in the host countries. In this scoping review and commentary, we describe the health literacy issues of FSU immigrants and provide an overview of FSU immigrants’ health literacy based on the integrated health literacy model. We purposefully consider the three most common locations where FSU immigrants have settled: the USA, Germany, and Israel. For context, we describe the healthcare systems of the three host countries and the two post-Soviet countries to illustrate the contribution of system-level factors on FSU immigrants’ health literacy. We identify research gaps and set a future research agenda to help understand FSU immigrants’ health literacy across countries. Amidst the ongoing global population changes related to international migration, this article contributes to a broad-scope understanding of health literacy among FSU immigrants related to the system-level factors that may also apply to other immigrants, migrants, and refugees.

## 1. Introduction

Health literacy has been defined as the ability and motivation of individuals to access, understand, process, and apply health information to make appropriate health decisions to optimize their health [1,2,3]. The concept of health literacy lies within the social determinants of health-conditions in which people are born, grow, live, work, and age [4,5].

Immigration status is an important social determinant of health associated with access to healthcare, health outcomes, self-rated health status, and health literacy [6,7,8,9]. Currently, few countries provide quality and accessible health services to the world’s 272 million international migrants [10]. Migrants and other people with low proficiency in a nation’s language often have limited health literacy [8]. Among immigrants, the culture, and social context within which people live can also contribute to health literacy [9]. Furthermore, the health of immigrants varies greatly and ranges from worse to better than the health status of native-born populations [11].

Among the growing number of international migrants, more than 25 million are from the former Soviet Union (FSU) [12,13]. FSU immigrants usually share several noteworthy characteristics relevant to health literacy. They typically speak Russian, have experienced a shared historical legacy, and come from a Soviet or post-Soviet healthcare system [14,15,16,17,18]. Furthermore, they tend to retain their cultural and social distinctiveness [19,20] and place importance on their Russian cultural identity [21]. Importantly, the Soviet healthcare system was the same across all Soviet republics and very different from the Western healthcare delivery model [14,17]. In general, FSU immigrants are white [22] and well-educated [23,24] highly skilled workers [25]. The majority migrate with educational attainment higher than the native-born populations of the USA [26], Germany [27], and Israel [28]. Nevertheless, FSU immigrants report their health to be poor or fair more frequently than native-born populations in the USA, European countries including Germany, and in Israel [29,30,31,32,33,34].

Despite their large numbers, unique socio-demographics, and health status, there is a paucity of literature available about FSU immigrants’ health literacy. To begin to fill this research gap, we provide an overview of FSU immigrants based on the integrated health literacy model developed by Sørensen et al. [3]. This agenda-setting article first describes adult FSU immigrants who migrated to the USA, Germany, and Israel in the last 30 years. We examine FSU immigrants shared historical and healthcare context as well as the healthcare systems of two post-Soviet countries (Kazakhstan and the Russian Federation) and the top three countries where FSU immigrants have settled (USA, Germany, and Israel). We review similar health literacy relevant issues among FSU immigrants residing in the USA, Germany, and Israel. The team of researchers from five countries provides an international perspective on health literacy and health equity by depicting healthcare systems while highlighting important health literacy implications of each. By including post-Soviet healthcare perspectives from Kazakhstan and the Russian Federation in comparison with the Western healthcare perspectives fromUSA Germany and Israel, we consider the contribution of system-level factors on FSU immigrants’ health literacy. Following the healthcare systems overview, a discussion section summarizes factors that influence FSU immigrants’ health literacy throughout the different countries and settings. We conclude the article by setting a future research agenda. 

## 2. Methods 

This article follows the methods of a scoping review. A scoping review allows researchers to identify the nature and extent of research evidence, providing an assessment of the size and scope of available research literature along with a narrative commentary [35]. In this case, our goal was to identify and synthesize a research literature base and research concerns related to FSU immigrants found across a variety of countries and contexts. This scoping review is important for researchers who study immigrants from the FSU in a particular setting as well as those who study other immigrant groups and seek to determine the health patterns and needs of people who cross international borders and might want to consider the similarities and differences between their community of interest and FSU immigrants. This review could also illuminate areas of concern, interest, and future directions for the field of health literacy. Specifically, the team of researchers from five countries identified the relevant literature and concerns related to the health literacy issues of FSU immigrants in each one of their host countries. We then synthesized the information to identify lessons learned, gaps, and future research directions. 

## 3. Overview of FSU immigrants 

Several terms describe people who move from one country to another; thus, clarification of terminology is appropriate. According to the United Nations, international migrants or immigrants, are people who live outside of their native country’s border [10]. Based on the USA terminology, an immigrant or migrant is a person who migrates to another country, usually for permanent residence; a refugee is a person who has been forced to flee his or her country of origin and is unable to return to that country and who might have been granted refugee status in the country of asylum [36,37]. Additionally, according to the definition by the International Organization for Migration [38], the term migrant refers to “any person who is moving or has moved across an international border or within a state away from his or her habitual place of residence, regardless of (1) the person’s legal status; (2) whether the movement is voluntary or involuntary; (3) what the causes for the movement are; or (4) what the length of the stay is.” Some sources further categorize migrants into labor or work migrants, return migrants, displaced migrants, and others. For consistency, in this article, the term immigrant is used. It includes the aggregate of all FSU immigrants, migrants, or refugees who moved from their place of origin to live in another country permanently. Finally, multiple terms are used in literature to describe FSU immigrants reflecting their heterogeneity and multi-ethnicity: Slavic, Eastern European, Soviet, Russian, Belorussian, Ukrainian, Russian-speaking, and others. We refer to them broadly as FSU immigrants. 

### 3.1. Historical and Geographical Background

The Soviet Union, also known as the Union of Soviet Socialist Republics (USSR), existed for the majority of the twentieth century (1922–1991), covered a sixth of the world’s total landmass, and comprised a population of approximately 300 million people at the time of its disunion [22]. After the dissolution, 15 independent countries, referred to as FSU, emerged. These included the Baltic States (Estonia, Latvia, and Lithuania); Central Asia (Kazakhstan, Kyrgyzstan, Tajikistan, Turkmenistan, and Uzbekistan); Eastern Europe (Belarus, Moldova, and Ukraine); Eurasia (the Russian Federation); and Transcaucasia (Armenia, Azerbaijan, and Georgia). The Baltic States have since joined the European Union (EU); others, such as the Commonwealth of Independent States, maintain ongoing alliances.

A verifiable count of FSU immigrants is difficult to estimate for several reasons, including changes in international borders since the USSR dissolution, which led to the changes in the tracking [39]. Furthermore, FSU immigrants are diverse ethnically and linguistically, with many speaking not only Russian but also their national language [16]. Shortly before the Soviet Union dissolution, according to the 1989 data, over 25 million ethnic Russians [40] and more than 30 million people who spoke Russian as their first language lived outside of the Russian Soviet Federative Socialist Republic (RSFSR) [16]. Today, an estimated 25–30 million FSU immigrants live outside of their countries of origin [12,13]. Table 1 describes FSU immigrants to the USA, Germany, and Israel.

### 3.2. Emigration to the USA, Germany, and Israel

Various historical and economic events contributed to the vast out-migration from the FSU. Only about 1%–2% of the Soviet population was living in poverty in the period 1987 to 1988 [47]. After the dissolution of the USSR, by 1993, the poverty rate increased substantially in all 15 newly independent countries; poverty reached 50% in Russia, 65% in Kazakhstan, and 26% in Ukraine [47]. By 2000, almost 11 million people left Russia, 4 million left Kazakhstan, and 6 million left Ukraine [48].

The FSU immigrants emigrated in a series of waves. While some were able to leave the Soviet Union as early as the 1940s, the vast majority left after USSR dissolution in 1991 [49]. Researchers from Russia describe the following periods of post-Soviet migration: (1) the first wave (1991–1998) emigrated for family reunification, for ethnic and religious reasons as asylum-seekers as well as for economic reasons, leaving a problematic socioeconomic environment in search of better opportunities; this period also prompted marriage-based emigration, (2) the second wave (1998–2008) of emigration included those who left for employment and investment opportunities, while asylum-seeking, family reunification, and marriage-based applications continued, and (3) the third wave (2008–2017) continued family reunification emigration and included the increasing number of those leaving for employment, business, economic, and educational opportunities often residing in the host country seasonally and some living in two countries [50].

The top three FSU countries from which people emigrate to the USA are the Russian Federation, Ukraine, and Uzbekistan [39]. In the USA, the majority of FSU immigrants are legal immigrants who came as asylum seekers, Diversity Immigrant Visa (“green card”) recipients, and through family-sponsorship or being an immediate relative of USA citizens [39,49,51,52]. Asylum seekers included Jews, Pentecostals, Armenians, and other minorities [25,49,50]. Between 1991 and 1999, almost half a million FSU immigrants arrived in the USA based on a special refugee status made possible by the Lautenberg Amendment [49]. Immigration status is important because newcomers to the USA with refugee status may be eligible for medical benefits, health insurance, government funding for transportation and resettlement, and other social benefits [25,53]. This influx of FSU immigrants led to the Russian language becoming the seventh most commonly spoken language at home, estimated at one million people [41]. Unofficial sources claim the number of Russian-speakers in the USA to be almost seven million people [42]. Other sources note that between 1991 and 2003, more than 1.3 million FSU immigrants settled in the USA [54]. Almost three million designated themselves as having a Russian ethnicity on the 1990 USA Census, but some of these people were born in the USA [12]. 

Israel and Germany, in addition to general asylum and work immigration policies, have immigration and citizenship policies based on the ideological obligation to support those who are Jewish or ethnic Germans wanting to return to their “fatherland” [55]. Thus, in Germany, there is a subgroup of immigrants named *Aussiedler* (ethnic Germans, many of them are from FSU countries, who resettle in Germany), and in Israel, they are called *Olim* (one who ascends) [21,55]. Germany, in addition to *Aussiedler* policies, provided special settlement programs for FSU immigrants who declare they are Jewish [56]. Importantly, *Aussiedler* group is often described separately in statistics and research, as they receive privileges such as German citizenship and unrestricted access to German social and healthcare systems upon arrival [55]. As ethnic Germans, they often have better German language proficiency than other FSU immigrants [55]. 

Most of the German FSU immigrants came from the Russian Federation (1.4 million) and Kazakhstan (1.3 million) [27]. According to the current census data, 2.7 million FSU immigrants in Germany are first-generation immigrants making FSU, and its successor states one of the most important regions of origin for immigrants to Germany [27]. Overall, more than half (57%) of FSU immigrants emigrated to Germany for family reunification, almost a tenth (8%) for work, and 7.9% arrived as refugees or asylum seekers [27].

In Israel, FSU immigrants are the largest and most significant immigrant group estimated at one million people comprising a prominent proportion of the total population [18,21,28,55]. The vast majority of FSU immigrants to Israel arrived based on their Jewish heritage (Law of Return) and family reunification [49,55]. The majority came from Ukraine and the Russian Federation [57]. About 30% of FSU immigrants to Israel are not Jewish; thus, not eligible for the full set of immigration benefits [58]. The main reasons FSU immigrants decided to move to Israel include the following: desire to ensure their children’s future, to follow a family member, and to live as a Jew in the Jewish State [59]. Although many FSU immigrants migrated out of perceived need, about a quarter report being not satisfied with their life in Israel [21,60].

### 3.3. Health Literacy Relevant Issues Among FSU Immigrants in the USA, Germany and Israel

Noncommunicable diseases (NCDs) and mental health conditions appear to be prevalent in this group. Among various factors, health status before emigration and degree of acculturation in the host country could contribute to the poorer health and higher disability of FSU immigrants compared to the native-born population in all three countries [29,30,31,32,33,34]. In Germany, FSU immigrants have higher rates of obesity and metabolic syndrome compared to the native-born population [61]. In the USA, FSU immigrants have the highest rates of hypertension compared to all other immigrant groups [62]. In both Germany and the USA, they have higher diabetes prevalence than the native-born population [63]. In Israel, FSU immigrants are more obese and commonly experience musculoskeletal, cardiovascular, and gastrointestinal disorders as well as two or more diseases; they have higher rates of alcohol use and homelessness; they also experience high levels of anxiety, depression, sensitivity, and hostility associated with challenges of integration into the new society and high expectations of professional and economic growth upon arrival to Israel [18,64]. For illustration purposes, in one study in an urban primary care setting in the USA, more than a quarter of FSU immigrants screened positive for a major depressive disorder (26.5%), and more than half were undiagnosed or untreated [65]. In Europe and North America, FSU immigrants present to health providers with psychiatric and psychosomatic symptoms [66,67] and some experience significant vulnerability to stress, posttraumatic stress, and depression symptoms [68]. Social alienation and acculturation levels influence their symptoms of depression [69]. Despite that, many FSU immigrants do not seek any mental health services [70]. Besides NCDs and mental health conditions, the prevalence of some communicable diseases is high among FSU immigrants. In Israel, FSU immigrants comprise about a quarter of all estimated drug users and present with higher rates of Hepatitis C, tuberculosis, and HIV/AIDS than Israeli-born drug users [71]. In Germany, researchers raised a public health concern regarding HIV/AIDS education for FSU immigrants [72].

Healthcare utilization, as well as health promotion and disease prevention activities among FSU immigrants, are also challenging. Cultural beliefs, health beliefs, prior experiences, and language proficiency of the host country contribute to the lower likelihood that FSU immigrant women and older people (less literature is available about male FSU immigrants) choose to participate in preventative activities such as diagnostic screenings [73,74,75,76]. In the USA, FSU immigrants are less likely to immunize their children [77]. Although older FSU immigrants have multiple chronic conditions, they seldomly engage in preventative actions such as physical activity [78] and may lack the skills to select healthy foods due to unfamiliarity with the foods and food labels in the host country [54]. In Germany, FSU immigrants are less compliant than native-born people with follow-up care, a phenomenon associated with dissatisfaction and difficulty communicating with health providers [79]. On the other hand, FSU immigrants can also over-utilize healthcare services [80], suggesting a lack of understanding of how healthcare systems in their host countries operate. Furthermore, providers who practice within a healthcare system that implies patient autonomy and individual responsibility for disease management have referred to older FSU immigrants as “unmanageable” patients [81]. They have also been called “difficult” patients due to language, cultural, and communication barriers [82]. FSU immigrants appear to be passive participants and contributors to their health, and their healthcare expectations may arise from previous healthcare experiences and assumptions that health professionals will take the leading role in their care [74,80,82,83,84,85]. Furthermore, FSU immigrants residing in the USA are more likely to participate in drug importation by utilizing the internet to fill prescriptions and find health information [86]. 

Additionally, all immigrants may experience challenges in changing their socioeconomic status, which is associated with health literacy. For instance, when compared to those with equal education, studies conducted in Israel in the 1990s, found that FSU immigrants were usually able to close earnings gaps with similarly aged native-born people within a period of 15 to 17 years; studies conducted more recently showed that although the wage gap between FSU immigrants and natives narrowed over time, inequality remained even after two decades in Israel [28]. When comparing Germany and Israel, FSU immigrants in Israel tend to be more economically successful than those in Germany [87]. In the USA, FSU immigrants reach earnings assimilation faster than in Israel [88]; however, female FSU immigrants in the USA are more likely than other white immigrant women to be unemployed, receive public assistance, and if working, to have lower occupational prestige [25]. FSU immigrants, like other immigrants, may experience challenges applying their foreign education credentials, degrees, and work experience in their host country’s labor market, which impacts their income, ability to access care, and health literacy applicable to the settings of the host country.

## 4. Healthcare Systems: Implications for FSU Immigrants’ Health Literacy

System-level factors influence individuals’ health literacy and, as such, they also link to health outcomes [3]. The integrated model of health literacy developed by Sørensen et al. [3] depicts the dynamic and complex perspectives of health literacy. The model incorporates factors influencing health literacy and the pathways that link health literacy to health outcomes. Some of these factors are individual-driven, while others are dependent on the healthcare system. The model is grounded in the empowerment of citizens to manage disease, health, and health risks [3].

The following sections provide an overview of healthcare systems across the three host countries and the two post-Soviet countries as an opportunity to consider how different healthcare systems may influence immigrants’ health literacy. We first consider the Soviet model for background and comparative purposes. Although the healthcare systems of the Russian Federation and Kazakhstan are not identical, they share a common legacy based on the Soviet healthcare system. For context, Kazakhstan (the Republic of Kazakhstan) is one of the largest and fastest-growing post-Soviet economies in Central Asia and was the first FSU country to perform health literacy assessment of the population. The Russian Federation is the largest by territory and population among all FSU countries and is in the process of analyzing the first health literacy assessment of the population.

### 4.1. Background: Soviet and Post-Soviet Healthcare Systems of Kazakhstan and the Russian Federation

To understand the contribution of system-level factors on FSU immigrants’ health literacy, it is necessary first to consider the original Soviet and current post-Soviet healthcare systems. Throughout the USSR, 15 Soviet republics used the Semashko model of primary care, named after the USSR Minister of Health Nikolai Semashko [89]. The government, based on the centralized planning and administration, financed the provision of basic services through publicly-owned healthcare facilities which were universally accessible and free at the point of delivery across dispersed populations [90,91]. Thus, all medical workers were public employees, and private practices were not allowed [89,91]. The “standardized planning norms” distributed services while aiming to reach the entire population [90] (p. 421). There was a large number of sub-specialists and a vast network of primary care, which was delivered through polyclinics (a collection of outpatient physicians serving patients in one location of a particular area) across urban areas and feldshers (community health workers) in rural areas [90]. Such an extensive and accessible health coverage addressed the population needs of that time and allowed for significant health improvements such as a decrease in infant mortality and communicable diseases, universal childhood immunization, and an increase in life expectancy; however, later, it also led to challenges related to inefficiency and economic burdens due to the high number of hospitals and hospital beds required and the longest hospital stays of all European countries [90]. 

This model suited previous population needs, such as control of communicable diseases and immunizations, but it was not adequate for the current challenging conditions such as NCDs [91]. For example, the communicable disease approach did not direct resources towards preventive healthcare services [17]; thus, in the USSR, the public healthcare system was underfunded and dominated by costs for inpatient care and a lack of incentives for better performance for health providers [89]. Nevertheless, the extensive coverage and universal access to free care provided an equitable system, although there were some qualitative differences between geographical regions [91].

Since the dissolution of the Soviet Union, this public health system has undergone many changes. Although the Russian Federation and Kazakhstan have undertaken significant efforts in reforming their post-Soviet healthcare systems, the is still an emphasis on the older administration and the centrality of the state [14]. The number of private providers, especially in the specialty care areas, has increased dramatically, offering services for out-of-pocket payments [89]. However, this increase comprises a small percent of publicly funded care [89]. A shift towards a more Western primary care model has been in development since the early 1990s; nevertheless, people still access primary healthcare through polyclinics, which are heavily underfunded since most of the funding goes toward inpatient care [89]. Changes in health service provision included a reduction of the hospital sector; however, the unnecessarily large inpatient sector continues to consume the bulk of health financing [92,93]. Underfunding creates inadequate infrastructure with long wait times and rationing of services [89]. The unavailability of robust primary care leads to people being hospitalized or utilizing emergency care to address complications of NCDs [92,93]. With both countries now offering private out-of-pocket services, some patients are opting to utilize private care to avoid long wait times. Since the dissolution of the USSR, there has also been a trend toward a less equitable distribution of medical services with people of a higher socioeconomic level having access to more and better services [92,93]. For example, in Kazakhstan, about 8% of people reported avoiding healthcare services due to the high costs [92], and the public health system is experiencing fundamental political and organizational challenges coordinating different agencies to address population health issues [94]. 

#### Notable Health Literacy Implications of the Post-Soviet Healthcare Systems

The Health Literacy Survey 47-item version developed by the European consortium (HLS-EU-Q) [95] was administered in Kazakhstan in 2013 (translated into Kazakh and Russian languages). Results revealed that a considerable proportion of the adult population of Kazakhstan had inadequate (15.5%) or problematic (30%) health literacy [96]. Higher general health literacy was associated with higher social status and the ability to afford medications, a low frequency of watching health-related TV programs, moderate community involvement, younger age, being married, and other factors [96,97]. In the Russian Federation, the Health Literacy Survey (HLS) 47-item version in the Russian language, which is somewhat different from the previously used version in Kazakhstan, is currently being analyzed to assess the health literacy of the adult population.

The results of the study in Kazakhstan prompted a call to action to improve population health literacy, to facilitate better communication skills of health providers, to organize health education resources, and to provide reliable health information to the population in plain language [96,97]. Almost 70% of participants in Kazakhstan reported having a university education, but the study did not find a correlation between educational attainment and the level of health literacy [96]. While an association between educational attainment and health literacy has been reported [98], educational attainment itself does not typically translate into high levels of health literacy [99]. The findings from Kazakhstan raise questions concerning whether the experience of participants with the post-Soviet healthcare system could have influenced participants’ answers to questions that were developed based on the Western healthcare systems. The relationship between the prevalent inadequate and problematic health literacy of the population and educational attainment is also unclear. 

### 4.2. Healthcare System of the USA

The USA health system is demonstratively different from the Semashko model. While the USA healthcare system has contributed significantly to medicine through numerous biomedical and technological advancements and accomplishments, it is also a system with a lack of organizational coherence [100]. Despite spending almost 18% of its gross domestic product (GDP) on health, the USA ranks last among 11 developed nations in overall health system performance [100,101]. Quality of care and access to care disparities exist and are associated with income, race, ethnicity, and gender [102,103]. 

The USA is the only developed nation that does not provide universal health coverage [100,104,105]. About a tenth of the population has no health insurance coverage of any kind [101]. The primary source of health coverage is private health insurance (64.2%) or public health insurance (37.7%), with few people having more than one type of coverage [106]. The public coverage includes Medicare for people age 65 or older, younger people with disabilities and people with end-stage renal disease (17.2%), Medicaid for low-income people, families with children, pregnant women, elderly, and people with disabilities (19.3%), or military healthcare (4.8%) [106,107]. Health insurance coverage through employment is often not continuous and usually changes every time a person changes their employer [100]. Despite the attempt to move towards universal coverage implemented by the Affordable Care Act (ACA), 27 million Americans remain uninsured and lack full access to the health services necessary to maintain and promote health [108]. 

Immigration status also affects access to USA health insurance since undocumented immigrants do not qualify to obtain a health insurance plan [109]. The total number of uninsured immigrants is unknown, but undocumented immigrants comprise about 12 million people [110]. If an individual is undocumented, does not have health insurance, or is unable to pay, then medical care is obtainable through a non-profit hospital emergency room or through a small number of community health centers that provide services free of charge or on a sliding scale [111]. Some immigrants may not seek medical care because they fear that accessing the medical system will compromise their ability to remain in the country [11,112]. Contrary to this belief, USA immigrants contribute more to the healthcare system than they utilize [113,114].

#### Notable Health Literacy Implications for FSU Immigrants in the USA

The term health literacy was introduced in the USA almost half a century ago [115], but it was not until the 2003 National Assessment of Adult Literacy (NAAL) assessed health literacy that the high prevalence of low health literacy in the USA became apparent. More than a third or about 36% of the adult USA population demonstrated basic or below basic health literacy, which means a lack of skills necessary for functioning in the increasingly complex healthcare settings [98,116,117].

One key area for health literacy in the USA is health insurance literacy. The concept of health insurance literacy is defined as an individual’s “knowledge, ability, and confidence to find and evaluate information about health plans, select the best plan for his or her family for their own or their family’s financial and health circumstances, and use the plan once enrolled” [118] (p. 6). FSU immigrants coming from the centralized healthcare system with universal coverage are most likely unfamiliar with the concept of health insurance and may experience challenges accessing care. A recent study found that about half of the adult USA population reported having inadequate health insurance literacy and low confidence in using insurance to access care with more than half having limited knowledge of the additional out-of-pocket and deductible costs; non-USA citizens were more likely to have less knowledge about health insurance [119]. Health insurance skills are independent of translation services, which are available in the USA free of charge as part of the culturally and linguistically appropriate services at no cost to the patient [120]. A patient may or may not have an interpreter, but unless they have prior experience or health literacy skills, they do not know what questions to ask their healthcare provider via the interpreter. These issues can be more challenging for older FSU immigrants who often have more disabilities in comparison to the native-born USA population [32] and may have more difficulty integrating and finding employment. In 2010, before the ACA, almost a quarter (23.2%) of FSU immigrants were reported to have no health insurance compared to 14% of the native-born population [31].

Thus, health literacy is not just about understanding the English language, it is about having the skills, knowledge, experience, and resources on how to, first, obtain insurance and then how to navigate the USA insurance system to obtain adequate healthcare. FSU immigrants who come from the Soviet healthcare system may be very unfamiliar with and overwhelmed by the array of insurance choices and eligibility requirements. Given the complexity of the USA healthcare system and the low health insurance literacy of native-born populations, immigrants, including FSU immigrants, may not know what they qualify for and where to find reliable and clear resources.

### 4.3. German Healthcare System

The German healthcare system is a universal coverage system that is employment-based and provides continuous coverage, even in the case of a job change or stopping work for any reason [100]. This system covers almost the entire population (99.8%) [101]. Medical services are generally affordable, have short waiting times, and high levels of satisfaction with out-of-hours care [121]. There is a separation between public health services, outpatient care, and inpatient care. Independent physicians or local associations of physicians provide outpatient care. Although there is universal coverage and unrestricted access to all care levels, the quality of this care is inconsistent due to the division into statutory (non-profit, quasi-public corporation handling sickness funds comprised of employers and employees’ contributions) and private health insurances. Statutory health insurance contributions are dependent on income rather than risk, while non-earning spouses and children receive coverage without surcharges. About 88% of the population has statutory health insurance, and 11% has private health insurance [121]. This division creates one of the biggest challenges for the German healthcare system as it leads to health inequalities. Furthermore, like the USA, Germany is experiencing rising healthcare costs, with 11.3% of the GDP spent on healthcare in 2012 [101,121].

Immigrants who are in Germany legally usually have health insurance and thus are entitled to the same healthcare as the native-born population. Asylum seekers, however, have restricted access that only includes care for acute diseases and necessary medical or dental treatments. Vaccinations and necessary preventative medical check-ups are covered [122]. Pregnant asylum seekers and women who have recently given birth are entitled to medical and nursing help. 

The private health insurance in Germany may create additional barriers for immigrants as it includes materials with additional health coverage packages, which people have to be able to understand and apply to make appropriate health decisions. People of higher socioeconomic status who tend to have higher health literacy are more likely to utilize private health insurance. Furthermore, while virtually universal coverage makes health more equitable, the unrestricted choice of hospitals and health providers could lead to immigrants having challenges navigating among providers and facilities. Significantly, immigrants in Germany use emergency rooms more frequently, which may be related to their overall lower socioeconomic status, unfamiliarity with the inpatient and outpatient settings, or familiarity with a centralized healthcare system [123].

#### Notable Health Literacy Implications for FSU Immigrants in Germany

Levels of health literacy (via the HLS-EU-Q 47-item version) were first assessed in Germany (in one federal state) in 2011, results showed that 11% had inadequate health literacy, and 35% had problematic health literacy [124]. A nationwide, representative study in 2014 confirmed this and found that 54.3% of the population had inadequate or problematic health literacy [125]. Germany performed in the middle of the other seven participating European countries [124]. People of older age, people with low socioeconomic status, and high frequency of physician visits had lower health literacy scores [126,127]. Immigrants in general, but especially immigrants less than 65 years of age, had limited health literacy [125,126]. However, the results rely on a small study population and therefore summarize immigrants from different settings and countries. In Germany, there is a lack of detailed data about health literacy among specific immigrant groups, including FSU immigrants.

Therefore, it is important to examine health literacy and relevant features of FSU immigrants in Germany for several reasons. In a nationwide census conducted in 2018 in Germany, 64% of FSU immigrants spoke German at home, of those who did not speak German, 85% spoke Russian [27]. The average length of stay for FSU immigrants was 20.5 years; the average age at immigration was 26.6 years, which is older compared to other immigrant groups [27]. Fifty-one percent of FSU immigrants were married, which is slightly higher compared to other immigrant groups. Only 9% of the 1st and 2nd generation of FSU immigrants were married to native-born Germans. The poverty threat rate among FSU immigrants is 24%, which is slightly lower compared to other immigrant groups, but higher than the 11.4% poverty threat rate among the native-born German population [27].

Although FSU immigrants in Germany have a somewhat better formal educational level than the native-born population [27], educational attainment does not alleviate migration stress, adjustment, and acculturation challenges and the consequent effects on health [128,129]. High educational attainment does not always translate into high health literacy, and vice versa [99]. Thus, since 2015, health education is part of the school curricula across Germany, aiming to influence the health literacy of FSU immigrants, other immigrants, and native-born populations [130]. 

Currently, studies on language proficiency, length of stay, integration, social support, financial resources, and education, all related to health literacy, but they are discussed and studied separately and have not been linked to health literacy research in Germany. The impact of digitalization on health and health literacy has also become an important topic that will have implications on the health literacy of FSU immigrants. Another implication is the fragmented and complex healthcare system that is potentially more difficult to understand, orient, and navigate for FSU immigrants than for the native population.

### 4.4. Israeli Healthcare System

Since 1995, Israel provides universal health coverage according to the National Health Insurance Law that covers almost the entire population. The system receives funding through the combination of income-related health tax and government funds [131]. The share of national expenditure on health out of Israel’s GDP was about 7.4% in 2017, compared to 11% in European countries with health systems similar to Israel’s [132]. Participation in a health insurance plan is mandatory for all Israeli residents, entitling comprehensive healthcare as a fundamental right. Thus, all citizens in the country join one of the four competing national health insurance (NHI) organizations, known as Kupat Holim (similar to the Health Maintenance Organization in the USA), which are run as not-for-profit organizations and are prohibited by law from denying membership to any Israeli resident [131]. 

All residents are entitled to primary and secondary care visits, diagnostic and laboratory services, hospitalizations, including births, and discounts on prescription medications. Primary care physicians serve as gatekeepers for access to specialists, but overall access to care is good, and waiting times are usually short [133]. Nevertheless, even with comprehensive healthcare, four-fifths of Israelis (about 90% Jewish and 62% of Arabs) [134] expand their health service coverage by purchasing a supplementary insurance plan. Supplementary commercial coverage allows quicker access and better service but is more expensive [132]. 

Insurance eligibility is available through a residency in Israel and not citizenship. New immigrants, if unemployed, are entitled to one year of free health insurance. After the year, if still unemployed, immigrants, as well as unemployed citizens, are required to pay for the basic coverage [135]. Employers are required to enroll any foreign workers (whether documented or undocumented) in health insurance programs, whose range of benefits is similar to that of NHI. Nevertheless, some of the people who are living in Israel do not have health insurance, including undocumented migrants who are not working. Several services are made available to all individuals irrespective of their legal or insurance status. These include emergency care, preventive mother and child health services, and treatment of tuberculosis, HIV/AIDS, and other sexually transmitted infections [131]. 

In Israel, inequalities persist, despite universal coverage. Constraining the growth of private health coverage is one of the biggest challenges for the Israeli healthcare system. The needs of the Arab population and immigrants from FSU and Ethiopia receive particular attention [133,136].

#### Notable Health Literacy Implications for FSU Immigrants in Israel

The HLS-EU-Q 16-item survey was conducted in Israel in 2011 in Hebrew, Arabic, and Russian and revealed that about a third (31%) of the adult population had inadequate or problematic health literacy [137]. Out of the maximum score of 16, lower health literacy scores were observed among Arabs (12.7) and FSU immigrants (12.9) and the highest scores among Jews (13.5). Health literacy was associated with socioeconomic status, years of education, and self-rated health. In addition, since FSU immigrants tend to report to be less healthy than native-born Israeli residents, some researchers hypothesize that healthier FSU immigrants settled in Europe or the USA, while those with more health challenges settled in Israel due to the access to national health insurance [18,66]. 

In Israel, health literacy is a topic included in public health academic training. The National Council on Health Promotion, an advisory group to the Minister of Health, is currently in the process of promoting the development of a National Action Plan for Health Literacy [138] in an effort to address distinct health disparities and the socioeconomic divide [139]. One issue for health literacy that is gaining importance in Israel is the concept of digital health literacy as low health literacy and low digital health literacy are associated with poorer health outcomes. 

## 5. Discussion

We examined healthcare systems’ contribution to the health literacy of a specific immigrant group across three countries in the context of their prior experience with their native healthcare system. This scoping review highlighted issues that go beyond localized health literacy in order to consider global factors relevant to the increasing international migration. We hypothesize that previous experiences and exposures to a healthcare system influences the health literacy of immigrants in their host countries. As can be seen in these examples, FSU immigrants may experience health literacy challenges generally and particularly when trying to navigate the healthcare systems of the USA, Israel, and Germany. Such challenges would likely be applicable in many other host country systems not described here. Many FSU immigrants are most familiar with the “free of charge” healthcare system and access to care through centralized district-specific polyclinics to which they were directed for care at the discretion of the health provider. Both non-centralized health insurance and non-centralized mechanisms to care may be confusing to FSU immigrants, leading to health literacy challenges in accessing, understanding, appraising, and applying healthcare information in an acute and outpatient setting.

Furthermore, FSU immigrants may not understand the concept of health insurance, insurance benefits, and associated insurance terminology. They may be unfamiliar with the importance and utilization of preventative services such as screenings and regular checkups. They may have difficulty with referrals and navigating among and between providers, causing them to over-utilize or under-utilize health services based on access to care and perceptions of health needs. The experience or lack of interaction experience with health professionals, as well as the context of interaction influences peoples’ health choices. FSU immigrants with no language proficiency may especially experience difficulty communicating and explaining their health needs to providers. Even with the use of the interpreter, they would need to be familiar with the healthcare system to ask specific questions. A level of acculturation is also required so that FSU immigrants can advocate for themselves and operate in the patient-driven healthcare systems.

FSU immigrants move to their host countries with a set of different health beliefs, cultural beliefs, and prior experiences, which ultimately can influence their health outcomes at every point of engagement with the new healthcare system. Also, seeking care for preventative services and making navigational decisions about when to see a provider and who to see may be a challenge for this population. Due to the prior experience with the Soviet or post-Soviet healthcare system, FSU immigrants may have difficulty trusting unfamiliar systems. While FSU immigrants may be used to and expect a more paternalistic style of communication with health professionals, they also report a lack of trust in healthcare providers [84], which could influence their health literacy. They may also have limited trust in their native healthcare system, which may translate into a lack of trust in the systems of their host countries. FSU immigrants, especially of older age, have difficulty managing their conditions, communicating with providers and establishing rapport. They also experience frustration in trying to understand the underlying causes of their diseases as well as an inability to advocate for themselves, and they must rely on family members [66,83,84,140]. These challenges are associated with a lack of trust, confusion, and feeling overwhelmed by the healthcare system. Those who recently emigrated, or seniors, may not know the sources of understandable and trustworthy health information and choose sources from their countries of origin or unofficial sources that may not be relevant to their new context. Linguistic and communication challenges may further exacerbate all of these factors. FSU immigrants may be unfamiliar with the medical terminology of the host countries, whether due to a lack of language proficiency or a lack of experience in engaging with health providers, leading to compromised communications. Mental healthcare may be particularly challenging due to these factors, which is unfortunate as high rates of NCDs among FSU immigrants have been associated with acculturation stress [29,141]. These findings may help healthcare providers consider FSU immigrants and their health literacy to develop specific approaches that support societal integration and health-promoting activities among this group.

Coming from a system based on the notion that healthcare is a fundamental right accessible to all, FSU immigrants may then choose to return to their country of origin for medical care or medicine since it is more economical and more familiar. The level of FSU immigrants’ acculturation and degree of integration into the host country’s society plays a role in how they approach their healthcare. Since FSU immigrants appear to differ in their values from native-speaking patients [66], it is essential to understand and consider those values as they apply to their health and health behaviors.

Importantly, identified challenges are not unique to FSU immigrants only. Health literacy is prominently affected by socioeconomic status and the associated self-assessed health status [137,142]. It is dependent on many factors, including education quality and language proficiency, which is especially vital for immigrants [99]. These issues may also be useful when considering health literacy in other immigrants, migrants, and refugees. For instance, for FSU immigrants, as with many other migrant groups, it can be challenging to engage in research projects or identify in large numbers in population-based surveys for reliable data. Thus, it can be challenging to assess their needs.

## 6. Future Research Agenda

This scoping review’s findings indicate that FSU immigrants’ health literacy needs to be further assessed and examined in the context of immigrants’ host countries’ healthcare systems. To the best of our knowledge, no health literacy model specific to immigrants, migrants, or refugees exists. Such a model would include a multilevel structure consisting of individual, interpersonal, organizational, social, and policy levels and elements unique to immigrants as well as modifiable and non-modifiable factors. To assess the health literacy of FSU immigrants in different countries, using the same tool, would be important as it could allow researchers to learn of needs across countries and also identify possible interactions with context-specific effects that support or limit health literacy in individuals and communities. These international cross-country comparisons could also identify the specific health needs and topics of relevance to FSU immigrants across locations. Given the findings in this scoping review, we suspect this would include healthcare system access and utilization issues, given the unique nature of the Soviet-style health system. These could lead to meaningful interventions and recommendations for immigrants, healthcare providers, and healthcare system stakeholders. Future research should then pilot test interventions across communities and countries to see both overall and context-specific efficacy and other considerations. Thus, a future research agenda could include the following aims: (a) based on the health literacy concept, develop a health literacy model that is specific to immigrants/migrants/refugees, (b) assess the health literacy of FSU immigrants in different countries using a standardized assessment tool, (c) perform international cross-country comparisons, and (d) develop appropriate interventions and recommendations for immigrants, healthcare providers, and healthcare system stakeholders. 

## 7. Conclusions

Society plays a role in the availability of accessible and understandable health information [143]. Thus, societies must take a proactive approach to designing immigrant-friendly healthcare systems defined as “health systems that consciously and systematically incorporate the needs of migrants into health financing, policy, planning, implementation, and evaluation, including such considerations as the epidemiological profiles of migrant populations, relevant cultural, language and socioeconomic factors and the impact of the migration process on the health of migrants” [38] (p. 133). “Migrants should be looked upon as a resource and an investment in the future, not merely as a burden to society” [144] (p. 2). 

The literature suggests the importance of addressing health inequities through research and practice focused on decreasing the unnecessary healthcare complexity so that high health literacy skills are not required to optimize health [145,146]; developing health systems customized to the needs of people and communities [147], and integrating international comparisons to identify countries with effective health literacy policies and strategies [148]. The continuously complex demands of healthcare systems that all individuals have to meet [116,146], make it even more difficult for immigrants to optimize their health while navigating unfamiliar healthcare systems.

Ideally, in a health-equitable society, policy and practice would allow immigrants to have the same healthcare access and coverage as the native-born population. With the ongoing increase in the number of international migrants (immigrants) and the diversification of societies, responsive and proactive healthcare systems would provide health information in multiple languages and engage immigrants through cultural mediators who help them navigate within the new healthcare system and become contributing members in their host countries [136,149,150]. Any immigrant group would likely benefit from health literacy research and targeted interventions in line with the proactive approach of migrant-friendly healthcare systems. 

## Figures and Tables

**Table 1:** Estimated former Soviet Union (FSU) immigrant population, education, and language proficiency.

FSU Immigrants	USA	Germany	Israel
Estimated population	1–7 million ^1^ [41,42]	3.5 million ^2^ [27]	1 million [43]
Host country’s language proficiency	22% speak English less than well [41]	7% basic & 28% middle school German [44]	20% have no or weak mastery of Hebrew [45]
Percent with the highest level of education (percent of native-born)	55% Bachelor’s or higher (33%) [26]	34% academically oriented education (31%)^3^ [27,46]	53% academic education (48%) [28]

^1^ By the use of the Russian language, based on the official and unofficial sources. ^2^ 2.7 million first-generation and 800,000 second-generation. ^3^ German educational system uses different educational levels compared to the USA and Israel.
